# Melt Pool Changes Characterization in Laser-Processed H11 Hot Work Tool Steel Using Point-by-Point Scanning Mode towards LPBF Process Optimization

**DOI:** 10.3390/ma17184631

**Published:** 2024-09-21

**Authors:** Krzysztof Fryzowicz, Radosław Bardo, Rafał Dziurka, Jakub Kawałko, Grzegorz Cios, Andrzej Stwora, Piotr Bała

**Affiliations:** 1 Faculty of Metals Engineering and Industrial Computer Science, AGH University of Krakow, Mickiewicza 30 Av., 30-059 Krakow, Poland; fryzowic@agh.edu.pl (K.F.); bardo@agh.edu.pl (R.B.); dziurka@agh.edu.pl (R.D.); 2 Progresja, Żelazna 9 Street, 40-851 Katowice, Poland; 3 Academic Centre for Materials and Nanotechnology, AGH University of Krakow, Mickiewicza 30 Av., 30-059 Krakow, Poland; kubaka@agh.edu.pl (J.K.); ciosu@agh.edu.pl (G.C.); 4 Faculty of Mechanical Engineering, Cracow University of Technology, al. Jana Pawła II 37, 31-864 Krakow, Poland; andrzej.stwora@pk.edu.pl

**Keywords:** additive manufacturing, powder bed fusion, laser, point-by-point, hot work, steel, surface, nanoindentation

## Abstract

Additive manufacturing techniques employing laser-based metal melting have garnered significant attention within the scientific community. Despite a decade of comprehensive research on the fundamentals of these techniques, there still remain unexplored facets related to heat flux impact on metallic alloys’ properties. Particularly, the effects of point-by-point laser operation on melt pool formation in metallic materials still remain unclear. Thus, this study focuses on the implications of laser metal melting, particularly investigating a point-by-point laser mode operation’s influence on melt pool formation and its geometry in the phase-transformation-sensitive material H11 hot work tool steel. To examine the melt pool, singular laser tracks with various laser parameters were scanned across H11 sheet metal, which allowed for the elimination of layer-by-layer heat cycles’ influence on the melt pool’s microstructure. Samples were examined by means of metallography, revealing significant differences in the melt pool’s depth, influenced mostly by exposure time rather than volumetric energy density. Heat-affected zone effects were found to have a limited range and thus potentially marginal effects in layer-by-layer manufacturing conditions. At the same time, retained austenite concentrations near fusion lines have been found within melt pools, suggesting potential micro-segregation of the alloying additions. The results present guidelines towards laser melting processes optimization.

## 1. Introduction

Additive manufacturing (AM) technologies utilizing laser sources for metal melting have gained increasing attention from the scientific community in recent years. Researchers have primarily focused on optimizing processes to produce high-quality parts and developing new materials tailored to these technologies. While the fundamental aspects of these processes have been extensively investigated over the past decades [1,2,3], the diversity of technology variations still holds some fairly well-discussed aspects. However, the specific effects of point-by-point laser working modes on melt pool formation remain underexplored, particularly when dealing with materials sensitive to phase transformations, highlighting a critical gap in the current research. One of these areas still requiring a deeper understanding is how the point-by-point laser working mode influences melt pool formation. This becomes particularly intriguing when dealing with phase-transformation-sensitive materials, where variations in heat dissipation rates and the size of the liquid metal pool can significantly impact the resulting microstructure. It is important to note that, depending on the size and geometry of the melt pool as well as the varying depth of the melted pool, the properties in the top layers of the printed element can be controlled and potentially improved, especially in phase-transformation-sensitive materials.

Among materials exhibiting phase transformations during cooling, H11 (1.2323) hot work steel emerges as an intriguing research subject. Widely utilized in the tool industry, its phase transformations have been already well harnessed in heat treatment technologies. In this context, the industry’s heat treatment processes underscore how profoundly the material’s properties can change based on thermal conditions during cooling from elevated temperatures. This material’s changes arise from the transformation of alloy carbides within the steel’s microstructure and appearance of retained austenite in a hardened state. Such heat treatments are also utilized already for additively manufactured hot work tool steels [4,5]. Despite the progress in understanding these treatments, the interaction between point-by-point laser mode and phase transformations during additive manufacturing is not yet fully understood, representing a novel research direction with significant implications for controlling material properties. However, depending on the proportions of these phases within the microstructure, various plastic and hardness properties of the material can be expected, enabling tailored applications in specific tool contexts [6]. Additionally, the amount of retained austenite in the tooling steel prior to the heat treatment route can significantly influence the final properties of the part, making this topic even more relevant for advancements in the 3D printing domain.

Chosen for the purposes of this investigation, H11 steel is a fairly well studied steel in the realm of additive manufacturing, largely attributed to the tooling industry’s demand for manufacturing freedom offered by AM paired with high-strength materials. Nevertheless, challenges persist in its additive fabrication, primarily stemming from the occurring phase transformations during cooling from elevated temperatures. These transformations, when accompanied by specific thermal conditions obtained during the additive manufacturing process and related to cyclic cooling with rapid rates, give rise to residual stresses that lead to micro-cracks, ultimately compromising the quality of additively manufactured components [7,8,9]. These conditions depend on numerous factors, partially linked to process parameters such as melting speed or beam power; AM process conditions involving build platform preheating or build height; and, most notably, laser operating mode, affecting the energy introduced to the material workpiece. Investigating how these unique factors interact under less commonly employed point-by-point laser modes could reveal new pathways for optimizing the mechanical properties and microstructure of additively manufactured components.

Most additive manufacturing machines utilizing laser beams for metal melting predominantly employ lasers in a continuous operation mode. However, this mode of laser operation results in energy accumulation within the material as successive tracks are scanned on the same layer [10], necessitating careful consideration when selecting manufacturing parameters [11,12]. Systems designed to mitigate energy accumulation by algorithmically reducing power during track melting or when reaching the edge of the printed element are rarely employed. Conversely, some 3D printing machines, especially those dedicated to laser powder bed fusion (LPBF) technology, utilize the point-by-point laser mode, leading to different thermal conditions in the material. Notably, these differences manifest in higher cooling rates (crystallization) and an increased number of thermal cycles affecting previously crystallized material [13,14]. These additional thermal cycles may (and generally do) induce cyclic phase transformations, inevitably influencing the microstructure of the final parts. Furthermore, understanding the impact of point-by-point laser beam operating parameters on weld pool geometries and the size of the heat-affected zone is crucial for comprehending the microstructure evolution of phase-transformation-sensitive alloys during laser processing in PBF.

The microstructure evolution during laser processing derives from the laser–metal interaction that induces the solidification and crystallization processes. In the realm of LPBF, diverse solidification conditions—namely, the thermal gradient and cooling rate—can be attained through the control of laser processing parameters, mostly involving laser power and scanning speed (controlled in point-by-point laser working mode by exposure time). In the case of hot work tool steel, two solidification modes can be observed based on thermal conditions: primary δ-ferrite and primary austenite, both possible in LPBF processing observed with different cooling rates of a melt pool [15]. The solidification mode, along with the resultant solidification structure, plays a major role in shaping solid-state phase transformations during cooling and intrinsic heat treatment. In addition to that, the thermal and fluid flow fields, deriving directly from laser processing conditions, may induce changes in melt pool geometry and grain morphology [16]. Thus, the investigation of microstructures resulting from different laser processing conditions by means of their morphology and phase distribution for a hot work tool steel becomes another crucial aspect of the research. 

Consequently, this research aims to evaluate the influence of point-by-point laser beam operation on the geometry and microstructure of the melt pool in H11 steel. With the acquired knowledge of how melt pool geometry changes depending on the parameters, it is possible to more consciously influence the final properties of the prints and exploit the laser’s working parameters to enhance the top layers of 3D-printed parts. This research, therefore, not only addresses a critical gap in understanding the effects of point-by-point laser operation but also explores its potential to unlock new levels of control over material properties in additive manufacturing.

## 2. Materials and Methods

### 2.1. Samples Fabrication

**Figure 1:**
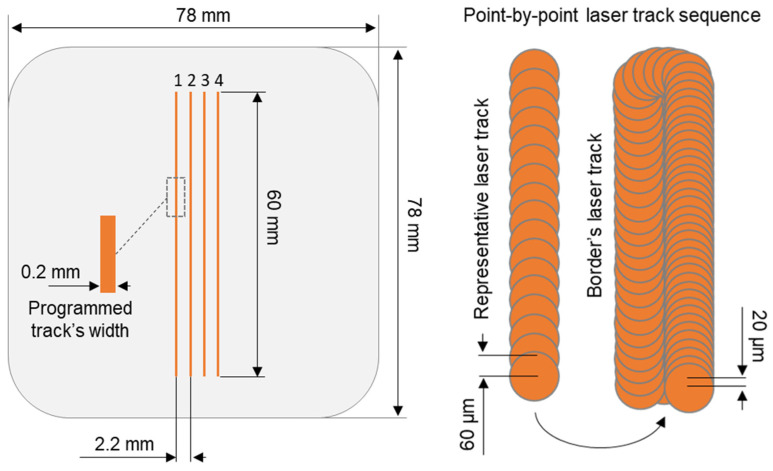
Laser tracks sequence visualization performed to obtain 4 representative laser tracks with point-by-point laser working mode giving the circular melt pools in every laser spot position.

In order to separate the effect of layer-by-layer manufacturing that causes the thermal cycles of processed material, the manufacturing experiment was planned to obtain several laser-melted tracks with different manufacturing parameters on the bulk (substrate) material (see scanning sequence on Figure 1). The substrate has been cut from the 5 mm thick H11 (1.2343, X37CrMoV5-1) annealed sheet metal with chemical composition presented in Table 1.

Laser tracks were scanned using Renishaw AM400 machine (Wotton-under-Edge, UK) dedicated to LPBF technology processes. Equipped with laser source with maximum output power of 400 W, machine’s final laser spot diameter was achieved at 70 µm with a laser wavelength of 1070 nm. The machine is distinguished by the point-by-point laser working mode, which gives us opportunity to control the laser speed by the parameter called laser exposure time. Since laser working in point-by-point mode stops in consecutive points during laser track scanning, the exposure time translates directly to the duration at which laser melts the feedstock material. Additionally, the machine’s software also allows for control of point distance parameters which specifies the distance between those laser stops. Melting process was performed under protective atmosphere of argon, with oxygen content below 1500 PPM. 

To achieve the representative laser tracks, the build job was programmed with rectangular 0.2 × 60 × 0.05 mm (200 × 6000 × 50 µm) objects, with 0.2 mm being width and 0.05 mm height (corresponding to expected layer thickness) spaced between each other by 2.2 mm. Due to the software’s build methodology, apart from laser tracks dedicated to volume hatching (in this case representative tracks) additional laser tracks are scanned accordingly to contour border. These additional tracks were programmed with constant for all sample parameters given in Table 2 and were scanned after the initial representative laser track. Variable manufacturing parameters used for laser processing of representative laser tracks have been given in Table 3. The volumetric energy density (VED) was simulated in order to evaluate impact of used laser processing parameters on expected LPBF process and calculated using of Equation (1) [13]:

VED = (P × e_t_)/(p_d_ × h_d_ × d) (1)

where P is laser power, e_t_ is exposure time, p_d_ is point distance, h_d_ is hatch distance, and d is layer thickness.

### 2.2. Sample Preparation

**Figure 2:**
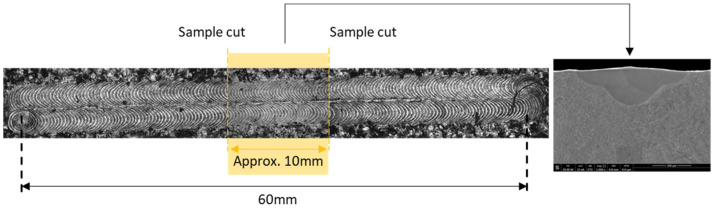
Laser tracks’ sample cut locations, with indication of sample cut for SEM analysis.

To prepare metallographic samples, the laser-scanned substrate plate was cut parallelly to laser tracks with cuts in between each track and with use of electrical discharge machining (EDM), which allowed for minimal cutting kerf. Then, each of four obtained longitudinal samples were cut twice crosswise to laser track axis, near the middle of the tracks with approx. 10 mm between each cut (See Figure 2). Thanks to this, approx. 10 mm long samples have been achieved with revealed cross sections of scanned laser tracks. 

Obtained samples were hot mounted in electrically conductive resin, then grinded and polished with use of standard metallographic route dedicated for hot work tool steels. 

### 2.3. Sample Examination

**Figure 3:**
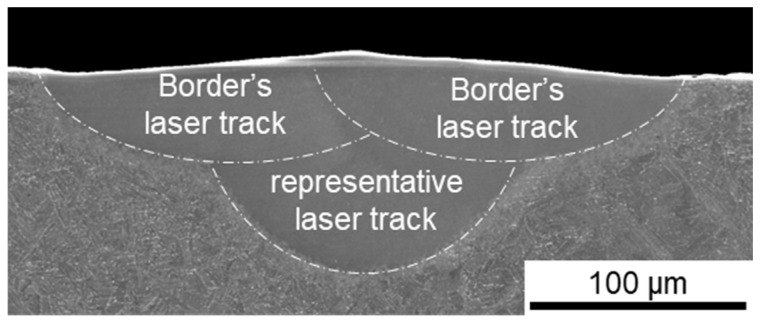
SEM SE image of cross-sectioned laser track with melt pool fusion lines indicated in white.

To observe the melt pool geometries of produced samples, light microscopy (LM) and scanning electron microscopy (SEM) were used. Firstly, the NIKON Eclipse MA100 (Tokyo, Japan), QIMAGING Micropublisher 3.3 RTV camera (Surrey, BC, Canada) was used to observe laser tracks’ surface in visible light (See Figure 2). Then, the Versa 3D FEI SEM (Hillsboro, OR, USA) was used to examine further the surfaces of laser tracks as well as cross-sectioned samples. SEM observations were conducted with use of secondary electron (SE) and backscattered electron (BSE) modes on samples etched with 10% oxalic acid (water solution). The image of cross-sectioned laser track observed in SE contrast are shown in Figure 2 and Figure 3. 

Electron back scatter diffraction (EBSD) maps were collected at an accelerating voltage of 20 kV and beam current of approximately 20 nA. EBSD Symmetry S2 camera manufactured by Oxford Instruments (Abingdon, UK) was used and operated in the speed 2 mode with effective pixel resolution of 156 × 128 and map pixel size of 100 nm. 

Nanoindentation was performed on the polished cross-sections, revealing the morphology of melt pools and heat-affected zones obtained in laser processing. The indentation was carried out using the Femtotools FT-NMT04 (Buchs, Switzerland) in situ nanomechanical testing system installed in FEI Versa 3D FEG SEM. A 200 mN load sensor and Berkovich indenter was used with area function calibrated on fused silica reference sample. The indentation was carried out in displacement-controlled mode to a fixed depth of 500 nm for all indents. Hardness values in MPa were obtained from indent contact area at maximum measured force and converted to HV Vickers hardness. For all tested laser tracks, an array of indents consisting of two line-scans was located to measure properties of substrate material as well as melt pool and heat affected zone. The indent and line-scan spacing in all tests was 5 µm.

## 3. Results and Discussion

**Figure 4:**
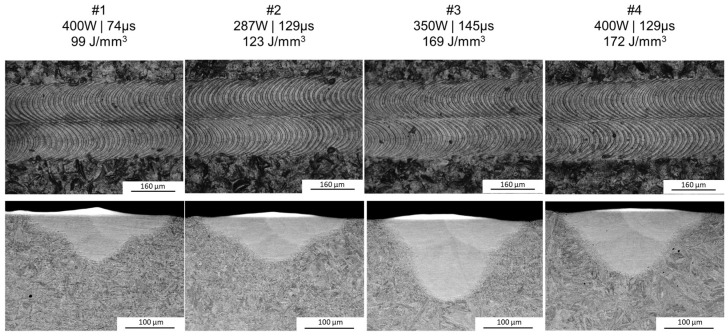
Cross-sections (**bottom**, SEM BSE) with the surfaces’ top views (**top**, LM) for all of the manufactured samples.

Since the border’s laser track melting parameters were constant throughout all of the samples and were scanned after the initial representative laser track, the LM of the tracks’ surfaces has revealed a similar topography (see Figure 4, upper photographs). When it comes to the SEM observations of the cross-sections, they have revealed that, indeed, the melt pool geometry has been significantly affected by different manufacturing parameters (Figure 4, bottom), with differences in both the depth and width of the melt pools.

**Figure 5:**
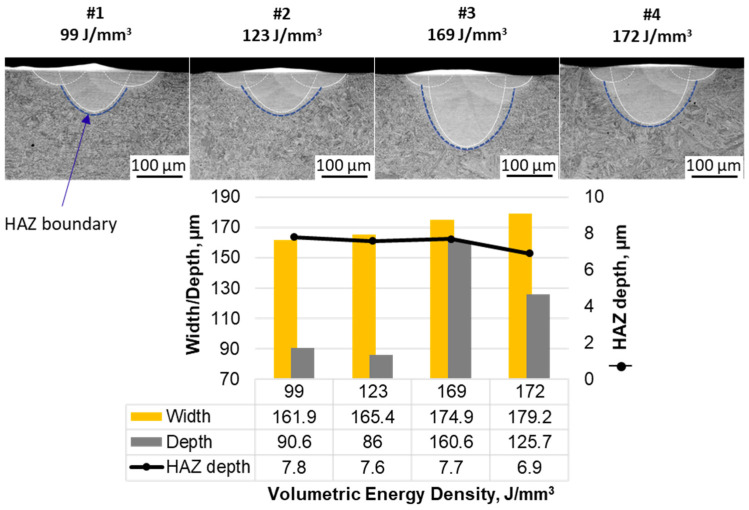
Melt pools’ geometry measurements with contours indicating HAZ boundaries.

The measurements of the melt pool geometry gave more insight on how the melt pool is affected by different manufacturing parameters. On the basis of Figure 5, the significant differences are seen mostly in the depth of the melt pool and the width/depth ratio. The most symmetric melt pool (approx. 0.9 depth to width) has been received for manufacturing parameter #3 that would introduce 169 J/mm^3^ of energy during LBPF volume hatching (350 W and 145 µs of exposure). This point, when compared to the #4 track (with a similar VED of 172 J/mm^3^ obtained with 400 W and 129 µs of exposure) might suggest that lower laser power with higher exposure time leads to a deeper melt pool. Also, an increasing trend is observed for the melt pool’s width with the VED. However, it seems that the VED itself does not influence the depth of the melt pools and the combination of both power and exposure time might be more crucial for this feature, especially since the samples with the highest and very similar VEDs differ in melt pool depth significantly. When it comes to the heat affected zone (HAZ), for all of the manufacturing parameters, similar HAZ depths were observed, showing a weak relationship of HAZ width and manufacturing parameters. The HAZ widths, however, are seen to be thicker than their depths. This is probably due to heat dissipation differences that are expected horizontally and vertically. Horizontally, there is only a limited volume in which heat can be dissipated, as other laser tracks are melted adjacently (2.2 mm) and the substrate plate is isolated, in this case by argon from elevator walls, which means that heat is not conducted any further in this direction. Vertically, on the other hand, the substrate plate is fixed to the 3D printer elevator platform. This allows for conductance of heat to the 3D printer’s chamber, which acts, in the case of singular laser tracks, as a heat sink, since several laser tracks’ heat can be easily dissipated in the volume of the chamber walls. This could explain why the HAZ depth and width is not of the same dimensions. Nevertheless, similar HAZs observed with different laser processing parameters can indicate that the manufacturing parameters used for 3D printing purposes would have had only limited influence on the previously deposited layers during the additive manufacturing process but could induce some microstructural notches between successive layers. Last but not least, the depth of the melt pools was largely above 50 µm (86 µm at the lowest), which suggests that all of the used parameters would have resulted in penetration of one (#1 and #2) or even two (#3 and 4) previously deposited layers (with an assumed layer thickness of 50 µm) during the AM process.

**Figure 6:**
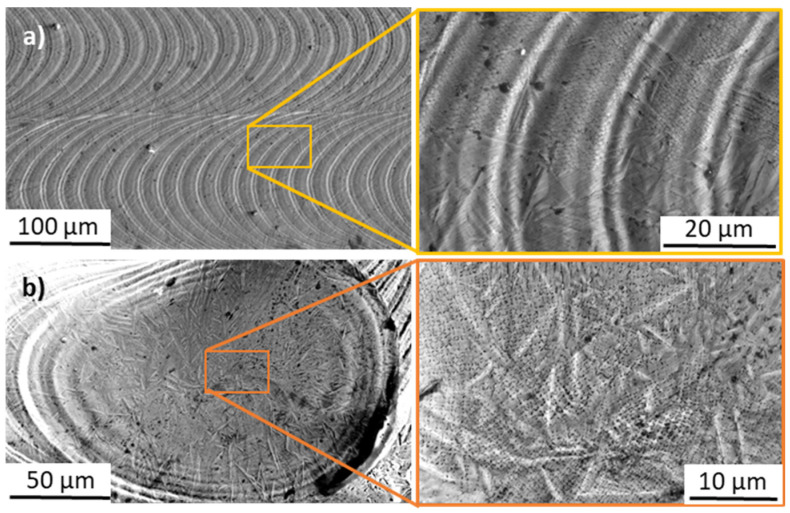
SEM observations of the sample’s surfaces (top view of a laser track), (**a**) in the middle of the laser track and (**b**) at the endpoint.

The samples’ surface observations with SEM (Figure 6) have shown the micro-dendritic microstructures (sub-micron dendrites with sizes below 1 µm) in successive laser interaction zones (known in welding technologies as welding scales). Microphotographs taken in the middle of the laser track (Figure 6a) show that successive melt pools were distant from each other by approx. 20 µm, which corresponds to the laser border manufacturing parameters (see Table 1). The same surface microstructures were seen in all of the examined samples resulting from the same laser border manufacturing parameters. When it comes to the laser track’s endpoint (Figure 6b), the micro-dendritic microstructure is visible with some elongated rod-like bright shapes going through multiple dendrites. Those shapes correspond probably to topographic anomalies within the surface of the laser track; similar bright areas are visible on Figure 6a in the boundary of successive melt pools, where molten metal could have been pushed radially by recoil pressure, or could evaporate axially to the laser beam, creating convex border edges. Finally, no preferred orientations have been spotted for the dendrites growing towards the axis of the melt pool from its boundary. 

**Figure 7:**
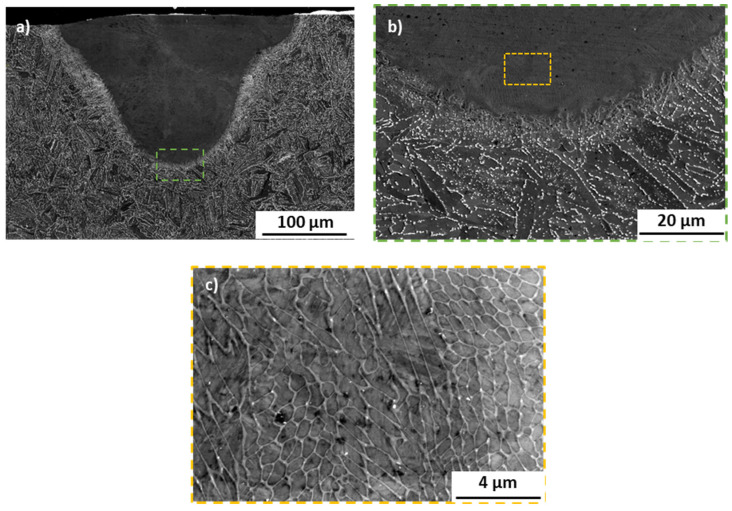
SEM microphotographs representing cross-sections of sample #3, taken with the BSE mode. Very similar microstructures have been observed for all of the remaining samples. (**a**) fusion line; (**b**) magnification of picture (**a**); (**c**) magnification of picture b in area of melt pool.

**Figure 8:**
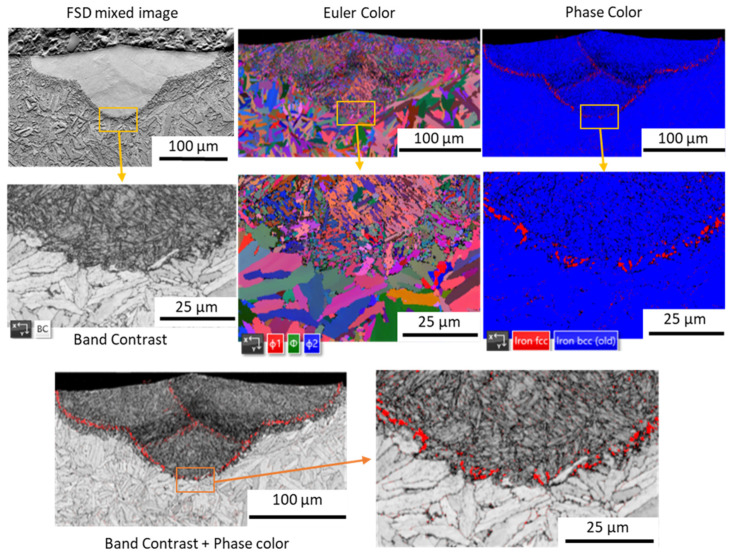
EBSD maps acquired for sample #3 with FSD mixed image, Euler color, phase color and band contrast.

The substrate microstructure has been revealed to be typical for annealed H11 hot work tool steel (Figure 7a), with carbides and retained austenite located at the grain boundaries (see also the EBSD map in Figure 8). This structure results from the material’s thermal history prior to laser processing, which involves slow cooling rates that allow for the formation and segregation of carbides and retained austenite. In contrast, the microstructure within the melt pool is significantly finer, without distinct grains or visible carbides at similar magnifications. This refinement is a direct result of the rapid cooling rates and high thermal gradients typically observed in LBPF processed steels; however, in the case of the used laser mode, this refinement can also be associated with the point-by-point laser scanning mode. The high cooling rates promote the formation of a cellular dendritic microstructure, which has been noted in other studies involving PBF processing of hot work tool steels [6,17,18]. The point-by-point exposure, on the other hand, might additionally refine the retained austenite due to additional thermal cycles introduced by the intermittent regime of laser energy release. No grain growth has been observed within the HAZs or any other regions in all of the examined samples, which may be expected in the laser melting process, typically characterized by small HAZ regions (in comparison to other welding methods). Also, no cracks were seen in the manufactured samples. This is especially interesting, since most of the research conducted with AM and hot work tool steels (H11 and the similar H13 grade) have shown a strong cracking tendency of hot work tool steels during the PBF process [8,19,20,21]. This might suggest that the formation of cracks in these steels is mainly caused by the cyclic character of layer-by-layer manufacturing, which imposes recurring thermal stresses on the processed material, rather than the hot-cracking mechanism governed by the alloying additions’ micro segregations [22]. These findings highlight the critical relationship between processing parameters, microstructures, and (in turn) the resulting properties of the material. The point-by-point laser mode not only creates a refined microstructure with fewer defects but also most probably reduces the tendency towards cracking, which can significantly enhance the mechanical properties and reliability of the produced components.

EBSD maps acquired for all of the samples have shown similar results to those presented on the representative sample #3 maps (Figure 8). The Euler colored map as well as the microphotograph showing the band contrast reveals the ultrafine microstructure of the melt pool in comparison to the substrate’s grained microstructure. A higher magnification of the Euler colored map shows that the orientation of the melt pool’s microstructure is not dependent on the substrate’s grains and no distinct texture can be seen in melt pool itself. Furthermore, in the melt pools’ microstructures, the columnar grains also cannot be distinguished, and only micron-sized dendrites were observed. Those dendrites seem to have no specific texture and are probably a result of the primary γ (austenite) crystallization character of the iron-based alloy with a moderate amount of carbon (0.4%wt). Firstly, a face-centered cubic (FCC) phase (austenite) crystallizes directly from liquid metal and then secondly, with a temperature drop, these austenite grains transform into a body-centered cubic (BCC) phase. With regard to the temperature drop rate, the FCC phase can be transformed into a diffusion-driven phase of ferrite, or, when high cooling rates occur, into martensitic-type phases (metastable phases) obtained due to the martensitic phase transformation. In the case of the investigated material, the cellular dendritic microstructure observed in melt pools proves the very high cooling rates of a liquid that must have initiated the martensitic phase transformations of primary austenite grains. Additionally, martensitic phase transformation is known for causing the formation of so-called retained austenite that is expected in interdendritic spaces [17,23], seen as white spaces in Figure 7c. 

The EBSD phase map acquired for the examined samples (Figure 8) presents difficulties with the identification of interdendritic spaces as a retained austenite due to their small dimension; however, it provides evidence of retained austenite concentrations in the melt pools’ fusion zones (Figure 8). Since these concentrations are also visible in the fusion zones of laser border tracks, this retained austenite must have been formed during crystallization rather than during cooling within HAZs. With this in mind, the formation of this phase at these locations could have been caused by crystallization front movement that was pushing the BCC phase, stabilizing the alloying additions of H11 (Cr, Mo, V) towards the substrate’s surface (but to a very short distance from the fusion zone) and changing the chemical composition of the area in the fusion zone to promote retained austenite formation. Additionally, it is provisioned that in the bottom of the weld pool, the cooling rate and temperature gradient must have been the highest [24], which also contributed to the retained austenite formation.

**Figure 9:**
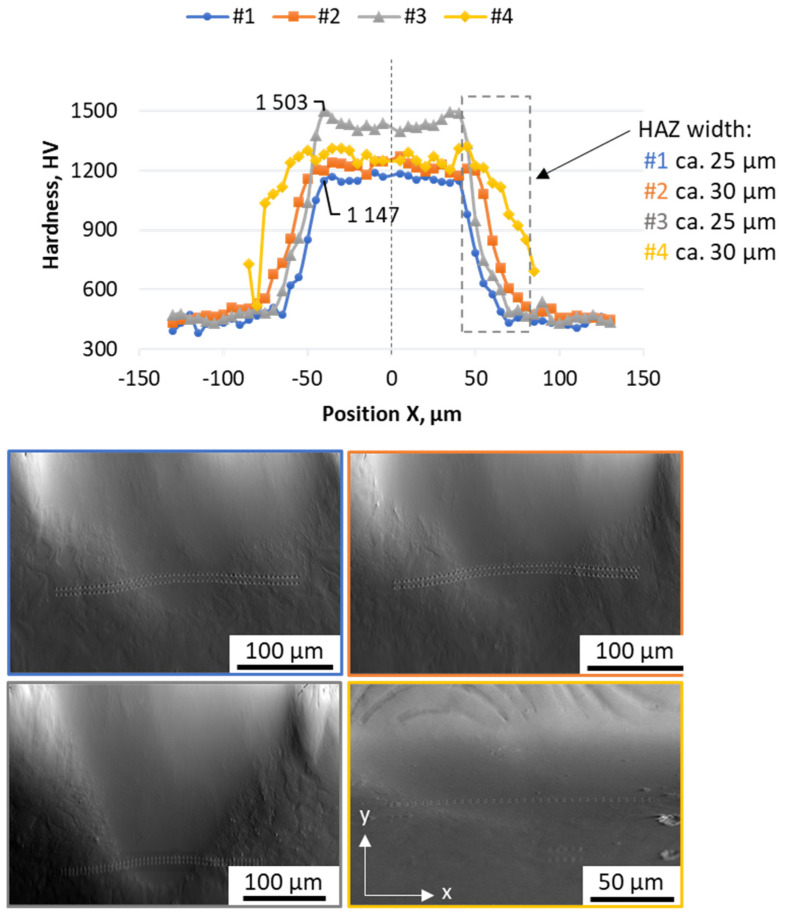
Hardness results acquired with the nanoindentation method for all of the produced samples. Results have been acquired with a left-to-right (horizontal) regime with two indentation lines. The presented measurements are acquired as a mean value from two indentation points at a given position.

Nanoindentation results (presented on Figure 9) have revealed the significant hardness differences between the melt pool and substrate material. Indentation line results show a smooth decrease of the hardness from the melt pools’ fusion line, confirming the relatively thin (with respect to the melt pool’s geometry) heat affected zones (HAZs). Due to these smooth transitions, the HAZ widths could have been estimated to be approximately 25–30 µm, 5–6 times wider than their depths. Also, this indicates that the microstructure in HAZs might be more tempered due to the effects of heat dissipation from the fusion lines. Still, the HAZ hardness was higher than the substrate plate (used in an annealed state). Since retained austenite was not found in the substrate plate with EBSD and is expected to be located mostly within the melt pool, this hardness might suggest the change in carbide composition that has increased the hardness of the HAZ (in regard to the substrate plate). This could also be achieved due to the quenching effect, but for that, the temperature in the HAZ must have been higher than the austenitization temperature (Ac1). In this case, however, the hardness would have change less smoothly, since the evident line (in the hardness changes curves) should have shown that it would divide the fresh martensite from the ferrite seen in the substrate plate. Since such a line is not seen neither in the hardness nor in the microstructure, this smooth hardness change could be attained via the tempering effect alone. Also, no retained austenite, which typically forms during quenching, was found within those areas, which further supports that theory. 

Where it comes to the hardness achieved within melt pools, different processing parameters resulted in significant differences, where sample #3 (169 J/mm^3^) had the highest measured hardness at approx. 1500 HV, whereas sample #1 (99 J/mm^3^) only had a hardness of 1150 HV (24% lower). This result shows that with laser processing parameters, the surface hardness of the material can be controlled. Additionally, sample #3, with highest hardness noted, also had the deepest melt pool (see Figure 5) at 160 µm. Such depth may allow for the application of laser processing to increase the wear resistance of 3D-printed objects by controlling the manufacturing parameters when reaching the top layers.

## 4. Conclusions

To conclude, the conducted experiment involved the fabrication of processing laser tracks representative for 3D printing using a PBF machine (equipped with a laser working in point-by-point mode) for the H11 hot work tool steel substrate. This has allowed us to investigate the relationship between the melt pool geometry and microstructure changes with different manufacturing parameters. Additionally, next to a representative laser track, an additional border track has also been scanned with the laser, just as it is in typical applications of the LBPF process. Samples have been investigated with LM and SEM and the following conclusions could be drawn from these observations:

- The melt pool’s geometry is influenced more by the exposure time and laser power than it is by VED. It is seen that parameters with a higher exposure and lower laser power were leading to deeper melt pools, whereas a VED increase can influence the melt pool only slightly.
- The microstructure of the laser tracks’ surfaces has revealed not only cellular dendritic microstructures but also the two-phase character of the crystalized melt pool, which must be related to retained austenite formation induced by the micro segregation of allying elements during the crystallization front movement. This has been confirmed by cross-section examination with SEM and EBSD methods.
- No cracking was observed in the melt pools, where a microstructure typical for PBF processed hot work tool steel was found, which may suggest that cyclic heating and cooling might play a more influential role in crack formation reported for this grade of materials.
- The clear evidence of a retained austenite-preferred location at the melt pool’s boundary (fusion line) was shown, resulting from the crystallization front movement towards the substrate plate surface accompanied by the fast-cooling rates occurring at the bottom of the melt pool.
- The heat affected zones observed in the manufacturing samples were thin in comparison to the melt pool geometries, which indicates only a limited influence of heat obtained during layer-by-layer manufacturing on the previously manufactured layers. This influence, however, might be more related to the remelting of previous layers due to the laser penetration depth.
- The hardness increase observed in melt pools might suggest the potential application of laser processing control in the top layers of a 3D-printed object to increase its hardness and, as a consequence, its wear resistance, as is similarly achieved in laser quenching (or laser hardening) processes.

The presented insights provide a basis for further optimization of the LPBF process parameters to enhance the quality of 3D-printed parts, especially for materials that are traditionally difficult to process due to their susceptibility to thermally induced cracking and microstructural changes. This contributes to a deeper understanding of the “processing–structure–properties” relationships in advanced materials, particularly in the context of additive manufacturing or other laser-involved processes. 

## Figures and Tables

**Table 1:** Chemical composition (%wt) of H11 sheet metal used as substrate in laser processing, investigated with Foundry-Master spark optical emission spectrometer.

C	Cr	Mo	V	Mn	Si	Ni	Cu
0.40	4.80	1.11	0.40	0.40	1.08	0.13	0.24

**Table 2:** Constant manufacturing parameters used for all produced samples. Layer thickness parameter has been added as typical value used in industry for steels to calculate the expected VED that would be achieved in LPBF process.

	h_d_ (µm)	p_d_ (µm)	d (µm)	P (W)	e_t_ (µs)	Expected VED (J/mm^3^)
Representative laser track	100	60	50	variable	variable	variable
Border	124 *	20	50	110	100	91

* considered border distance.

**Table 3:** Variable manufacturing parameters for singular laser tracks.

Sample No.	Laser Power (W)	Exposure Time (µs)	Expected VED (J/mm^3^)
1	400	74	99
2	287	129	123
3	350	145	169
4	400	129	172

## Data Availability

Not applicable.
